# Efficacy of temozolomide and bevacizumab for the treatment of leptomeningeal dissemination of recurrent glioblastoma: A case report

**DOI:** 10.3892/ol.2015.2940

**Published:** 2015-02-06

**Authors:** YOSHIKO OKITA, MASAHIRO NONAKA, TORU UMEHARA, YONEHIRO KANEMURA, YOSHINORI KODAMA, MASAYUKI MANO, SHIN NAKAJIMA

**Affiliations:** 1 Department of Neurosurgery, Osaka National Hospital, National Hospital Organization, Osaka 540-0006, Japan; 2 Division of Regenerative Medicine, Institute for Clinical Research, Osaka National Hospital, National Hospital Organization, Osaka 540-0006, Japan; 3 Department of Central Laboratory and Surgical Pathology, Osaka National Hospital, National Hospital Organization, Osaka 540-0006, Japan

**Keywords:** leptomeningeal dissemination, recurrent glioblastoma, bevacizumab

## Abstract

The prognosis of leptomeningeal dissemination of recurrent glioblastoma is poor, and chemotherapy results in minimal palliative efficacy. Temozolomide (TMZ) is an established therapy for patients with malignant glioma and the standard of care in parenchymal gliomas; however, few reports have been published with regard to its use for the treatment of leptomeningeal dissemination. Only one report has indicated the radiographic response of leptomeningeal dissemination to a TMZ rechallenge, suggesting a potential causative effect. While bevacizumab is an effective therapy for recurrent glioblastoma, its effect on leptomeningeal dissemination of recurrent glioblastoma remains unclear. The present study reports a case of leptomeningeal dissemination of recurrent glioblastoma in which transient neurological and radiological improvement was observed following chemotherapy with TMZ and bevacizumab. However, five months after the diagnosis of leptomeningeal dissemination the patient succumbed to the disease.

## Introduction

Glioblastomas are the most common type of common primary intracranial tumor. The prognosis of recurrent glioblastoma is poor with a patient survival time of between one and two years. Temozolomide (TMZ) is the standard therapy for glioblastoma patients. A recent study revealed an improvement in the median survival time from 12.1 months to 14.6 months upon the addition of concurrent TMZ to the previous standard therapy of surgery and radiotherapy in glioblastoma patients (1). In cases of recurrence, the median survival is approximately six months (2,3). Leptomeningeal dissemination, an end-stage complication of glioblastoma, is considered to be untreatable, with reported mean survival times of two to four months (4–6). Furthermore, previous reports have indicated that chemotherapy for leptomeningeal dissemination has limited therapeutic efficacy (4,7).

The utility of TMZ for the treatment of leptomeningeal dissemination of recurrent malignant glioma has not been determined, with only one study reporting a radiographic response to a TMZ rechallenge, which indicates a potential causative effect (8). Nandipati *et al* (8) presented the cases of two anaplastic glioma patients who exhibited durable responses of leptomeningeal dissemination to TMZ treatment. One patient received 15 TMZ chemotherapy cycles (200 mg/m^2^/day for 5 days in a 28 day cycle) in total with radiographic improvement after four cycles and resolution following six cycles of TMZ. The patient remains alive 34 months following the diagnosis of leptomeningeal dissemination. The second patient was treated with radiation therapy without concurrent chemotherapy and then a subsequent 12 cycles of TMZ chemotherapy were administered. All enhanced disease sites disappeared after six cycles. Nine months following the discontinuation of chemotherapy, neuroimaging revealed multiple, asymptomatic, enhanced nodules involving the leptomeninges. The patient received additional TMZ chemotherapy and all lesions disappeared after an additional six cycles. Forty-eight months following the initial diagnosis of leptomeningeal dissemination, the patient demonstrates no neurological deficit on TMZ (chemotherapy was ongoing at the final follow-up in 2010).

Bevacizumab, a monoclonal antibody that inhibits vascular endothelial growth factor (VEGF), is an effective established therapy for recurrent glioblastoma, following treatment with radiotherapy plus TMZ (9). Bevacizumab is also approved for the treatment of metastatic colorectal, non-small-cell lung, breast, ovarian and renal cancers. Although a pilot study demonstrated some response of stable disease to bevacizumab in a small sample of patients with leptomeningeal metastases from breast and lung cancers, in addition to melanoma (10), only one study has examined the effect on leptomeningeal dissemination of glioblastoma, and no improvement was observed (11).

The current study reports the case of one patient treated with TMZ and bevacizumab for leptomeningeal dissemination of recurrent glioblastoma. Written informed consent was obtained from the patient.

## Case report

**Figure 1:**
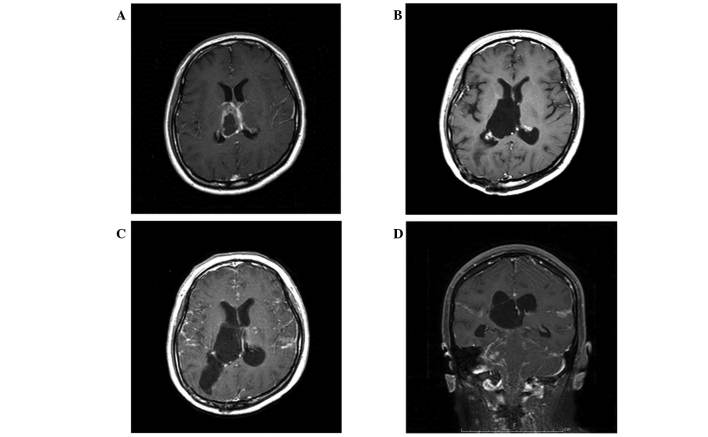
(A) MRI prior to surgery showed a tumor in the right thalamus enhanced with gadolinium diethylenetriamine pentaacetic acid. (B) MRI following maintenance TMZ therapy showed the enhanced lesion in the right thalamus had disappeared without recurrence. (C and D) Recurrent tumor was revealed on MRI; leptomeningeal dissemination was identified in the supra- and infratentorial brain without primary site recurrence. MRI, magnetic resonance imaging; TMZ, temozolamide.

**Figure 2:**
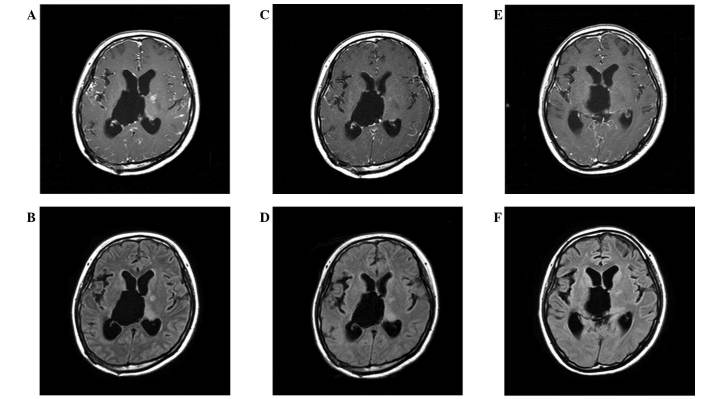
(A, B) MRI showed the progression of leptomeningeal dissemination following one cycle of TMZ and interferon-β therapy. (C, D) Leptomeningeal dissemination on MRI decreased following two cycles of TMZ and one cycle of bevacizumab from the beginning of recurrent therapy. (E, F) MRI showed no progression of leptomeningeal dissemination following three cycles each of TMZ and bevacizumab from the beginning of recurrent therapy. A, C and E: T1-weighted contrast-enhanced MRI; B, D and F: Fluid-attenuated inversion recovery imaging. MRI, magnetic resonance imaging; TMZ, temozolomide.

A 28-year-old female with glioblastoma was admitted to the Department of Neurosurgery, Osaka National Hospital (Osaka, Japan) after presenting with diplopia and numbness in the left hand. Magnetic resonance imaging (MRI) revealed an enhanced mass in the right thalamus (Fig. 1A). The tumor was surgically removed, and the histological diagnosis was determined to be glioblastoma. Radiotherapy (60 Gy/30 fr) with concurrent TMZ (75 mg/m^2^ per day) was administered for a month and a half, following which the patient was discharged from the hospital. A further 24 cycles of outpatient maintenance chemotherapy with TMZ were administered: First cycle, 150 mg/m^2^; second cycle onwards, 200 mg/m^2^, for the first five days of each 28-day cycle. No complications or recurrence were observed during maintenance chemotherapy (Fig. 1B). However, two months following the completion of the maintenance TMZ therapy, the patient experienced a seizure and disturbed consciousness. MRI revealed leptomeningeal dissemination in the supra- and infratentorial brain without primary site recurrence (Fig. 1C and D). Cerebrospinal fluid (CSF) cytology specimens were positive for malignant cells. Following one cycle of combined TMZ (150 mg/m^2^) and interferon-β therapy (3 MU) for the first five days of a 28-day cycle, MRI indicated progression of the leptomeningeal dissemination (Fig. 2A and B).

**Figure 3:**
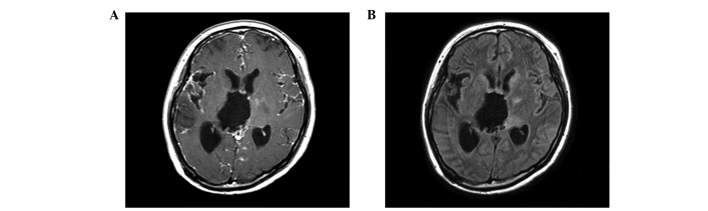
MRI showed progression of leptomeningeal dissemination following four cycles of TMZ and five cycles of bevacizumab from the beginning of recurrent therapy. (A) T1-weighted contrast-enhanced MRI; (B) fluid-attenuated inversion recovery imaging. MRI, magnetic resonance imaging; TMZ, temozolamide.

TMZ (150 mg/m^2^) for the first five days of a 28-day cycle and bevacizumab (10 mg/kg) for every day of a two-week cycle were administered. Following two cycles of TMZ and one cycle of bevacizumab from the beginning of recurrent therapy, the leptomeningeal dissemination observed on MRI decreased (Fig. 2C and D). The patient’s consciousness improved marginally, and she was gradually able to maintain simple communication. Following three cycles each of TMZ and bevacizumab from the beginning of recurrent therapy, MRI showed no deterioration of leptomeningeal dissemination (Fig. 2E and F). However, following four cycles of TMZ and five cycles of bevacizumab from the beginning of recurrent therapy, progression of the leptomeningeal dissemination was observed (Fig. 3), and the patient became bedridden. Palliative treatment was administered, and the patient succumbed to the disease progression one month after the TMZ and bevacizumab therapy was discontinued.

## Discussion

Leptomeningeal dissemination occurs in ≤20% of supra- and infratentorial glioblastoma cases, as described in several autopsy studies (4,12,13), with a mean survival time of two to four months (4–6). Radiotherapy and chemotherapy result in minimal palliative efficacy, without increased survival (6,14).

Bevacizumab is a humanized monoclonal antibody against VEGF, which has demonstrated improved progression-free survival (PFS) in phase III trials of metastatic breast (15) and renal cancers (16), in addition to prolonged overall survival in metastatic colorectal (17,18) and non-small cell lung cancers (19). Bevacizumab has also been used as a successful therapy in cases of leptomeningeal metastases from breast (20), non-small cell lung (21), and colorectal cancers (22). Furthermore, a pilot study (n=15) indicated that bevacizumab significantly decreases CSF VEGF levels over time, resulting in clinical, imaging and CSF responses, or stable disease in 54–73% of leptomeningeal metastases patients with breast cancer, lung cancer, or melanoma (10,23).

Chemotherapy as second- or third-line metastatic treatment has been established for other types of cancer, including breast and colorectal cancer (24,25). While the blood-brain barrier is disrupted by metastases, bevacizumab may aid in normalizing this disrupted tumor vasculature and improve drug delivery by facilitating uniform distribution of the vasculature (26–28). As a result, the use of bevacizumab has aided in prolonging the response to second- or third-line metastatic treatment in other types of cancer (20).

Other than TMZ or bevacizumab, no effective agent is available to treat glioblastoma. Bevacizumab alone or in combination with irinotecan was similarly effective for recurrent glioblastoma in the BRAIN study (29). Wong *et al* (30) reported that the six-month PFS rate and overall survival following bevacizumab therapy for recurrent glioblastoma were 45% and 9.3 months, respectively. However, the role of bevacizumab in the treatment of leptomeningeal dissemination of recurrent glioblastoma remains unclear due to the limited number of reported cases. The one previously published study did not report any improvement with this treatment (11).

TMZ is the standard therapy for patients with malignant glioma (1) and is considered the standard of care in parenchymal gliomas, but little published information exists with regard to the use of this agent to treat leptomeningeal dissemination. Nandipati *et al* (8) reported a radiographic response to TMZ rechallenge in patients with recurrent anaplastic glioma and leptomeningeal dissemination. More severe leptomeningeal dissemination was observed on MRI in the present case compared with those in the study by Nandipati *et al*; however, neurological and radiological improvement was observed following chemotherapy with TMZ and bevacizumab for almost 10 weeks. We hypothesize that bevacizumab may help to normalize disrupted tumor vasculature and improve TMZ delivery, providing increased CNS penetration.

In conclusion, the current case report indicates that chemotherapy with TMZ and bevacizumab may result in short-term improvement of leptomeningeal dissemination of recurrent glioblastoma, with demonstrated neurological and radiological improvement. Further, large-scale studies are required to investigate the effect of chemotherapy regimens with TMZ and bevacizumab on patients with a wider variety of malignancies.
